# Using Implementation Mapping to Build Organizational Readiness

**DOI:** 10.3389/fpubh.2022.904652

**Published:** 2022-05-12

**Authors:** Amber K. Watson, Belinda F. Hernandez, Jenny Kolodny-Goetz, Timothy J. Walker, Andrea Lamont, Pam Imm, Abraham Wandersman, Maria E. Fernandez

**Affiliations:** ^1^Wandersman Center, Columbia, SC, United States; ^2^Center for Health Promotion and Prevention Research, School of Public Health, University of Texas Health Science Center at Houston, Houston, TX, United States

**Keywords:** implementation science, organizational readiness, implementation strategies, implementation mapping, change management

## Abstract

Organizational readiness is essential for high-quality implementation of innovations (programs, policies, practices, or processes). The *R* = MC^2^ heuristic describes three readiness components necessary for implementation—the general functioning of the organization (general capacities), the ability to deliver a particular innovation (innovation-specific capacities), and the motivation to implement the innovation. In this article, we describe how we used the *Readiness Building System (RBS)* for assessing, prioritizing, and improving readiness and *Implementation Mapping (IM)*, a systematic process for planning implementation strategies, to build organizational readiness for implementation of sexual assault prevention evidence-based interventions (EBIs). While RBS provides an overarching approach for assessing and prioritizing readiness constructs (according to the *R* = MC^2^ heuristic; *Readiness* = *Motivation* x *general Capacity* × *innovation specific Capacity*), it does not provide specific guidance on the development and/or selection and tailoring of strategies to improve readiness. We used the five IM tasks to identify and prioritize specific readiness goals and develop readiness-building strategies to improve subcomponents described in the *R* = MC^2^ heuristic. This article illustrates how IM can be used synergistically with the RBS in applied contexts to plan implementation strategies that will improve organizational readiness and implementation outcomes. Specifically, we provide an example of using these two frameworks as part of the process of building organizational readiness for implementation of sexual assault prevention EBIs.

## Using Implementation Mapping To Build Organizational Readiness

Organizational readiness is important for effective implementation of any program, policy, practice, or process (1–4). An understanding of *how ready* an organization is can be helpful for organizations as they prepare to implement new interventions and throughout the process of implementation (4). However, the link between determining readiness and the actions needed to improve readiness has not been systematically described and there is scant literature to support specific evidence-based strategies for building readiness. A systematic approach linking readiness needs to actionable implementation strategies that are designed to build readiness can address this gap. In this article, we describe how we used Implementation Mapping (IM; see list of all abbreviations used in Table 1) to develop actionable readiness building strategies in an applied project to prevent sexual assault (5).

**Table 1:** List of abbreviations.

	**List of Abbreviations**
CMOR	Change management of organizational readiness
EBI	Evidence-based intervention
IM	Implementation mapping
ISF	Interactive systems framework
MSSAP	Multi-Site Sexual Assault Prevention Initiative
R = MC^2^	Readiness, motivation × innovation-specific capacity × general capacity
RBS	Readiness building system
RDS	Readiness diagnostic scale
TA	Technical assistance

Compilations of implementation strategies, such as the Expert Recommendations for Implementing Change [ERIC; (6)], are readily available to organizations and planners. What is limited, however, is specific guidance about which strategies to use (7). Additionally, even after strategies are selected, the content and details of those strategies (e.g., technical assistance, training) must still be developed. Researchers and implementers have had little guidance on how to improve critical implementation factors, such as organizational readiness, to achieve more effective implementation. They often select inappropriate strategies and/or struggle with the content of implementation strategies to improve readiness and implementation outcomes (7, 8).

**Figure 1:**
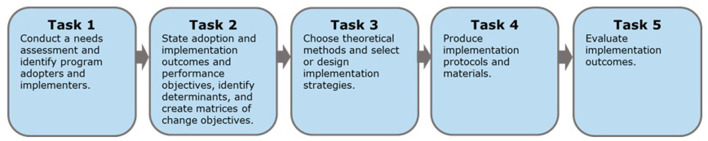
The five steps of implementation mapping [IM; (5)].

IM is a systematic approach for developing or selecting and tailoring implementation strategies to accelerate evidence-based intervention (EBI) uptake and use and increase the likelihood of sustainability. It includes a five-step process that incorporates implementation and behavioral science theories and frameworks, empirical evidence, and community and stakeholder input. IM clearly articulates implementation outcomes, actions (implementation behaviors), determinants, and expected outcomes, and it describes a process for developing targeted implementation strategies. By identifying and linking these elements, the IM process articulates the mechanism through which implementation strategies are intended to work. Recent studies have described its application to improve the implementation of EBIs in clinics, communities, and schools (9–11). The five steps are listed and discussed in detail in both Figure 1 and the Methods section (5).

### Readiness and the Readiness Building System

According to Nilsen (12) categorization, implementation science “determinants frameworks,” such as the Interactive Systems Framework (ISF) for Dissemination and Implementation can help identify the barriers and facilitators to implementing EBIs in new settings (13). According to the ISF and other frameworks, organizational readiness is a critical aspect (determinant) of successful implementation (14). The *R* = MC^2^ heuristic (Readiness = *M*otivation × Innovation-Specific *C*apacity x General *C*apacity), derived from the ISF, expands our understanding of organizational readiness and posits that each component is critical for successful implementation (4).

*Motivation* refers to the degree to which an organization wants and is committed to the implementation of the EBI. *General capacity* refers to the overall ability of an organization to function successfully on a day-to-day basis. *Innovation-specific capacities* are the abilities necessary to implement a specific intervention (program, policy, practice, or process) with quality. Each component has multiple subcomponents that are described in Table 2. A premise of the *R* = MC^2^ heuristic is that organizations must have sufficient capacities and motivation for successful implementation. Therefore, when motivation or capacities are low, additional efforts to build readiness are needed to ensure that an innovation (e.g., EBI.) will be successfully implemented.

**Table 2:** Readiness components and subcomponents.

**Subcomponent**	**Definition**
Motivation	Degree to which the organization wants the new innovation to happen.
Relative advantage	The degree to which the innovation seems more useful than what has been done in the past.
Compatibility	The degree to which the innovation fits with how the site does things.
Simplicity	The innovation seems simple to use.
Ability to pilot	Degree to which the innovation can be tried out.
Observability	Ability to see that the innovation is producing outcomes.
Priority	Degree of importance of the innovation in relation to other things the site does.
Innovation-specific capacity	What we need to implement the innovation.
Innovation-specific knowledge & skills	Sufficient abilities to implement the innovation.
Program champion	A well-connected person who supports and models the use of the innovation.
Supportive climate	Necessary supports, processes, and resources to enable the use of the innovation.
Intra-organizational relationships	Relationships within the site that support the use of the innovation.
Inter-organizational relationships	Relationships between the site and other organizations that support the use of the innovation.
General capacity	The overall functioning of the organization.
Culture	Norms and values of how things are done at the site.
Climate	The feeling of being part of the site.
Innovativeness	Openness to change in general.
Resource utilization	Ability to acquire and allocate resources including time, money, effort, and technology.
Leadership	Effectiveness of leaders at multiple levels.
Structure	Effectiveness at communication and teamwork.
Staff Capacities	Having enough of the right people with the right knowledge/skills, to get things done.

**Figure 2:**
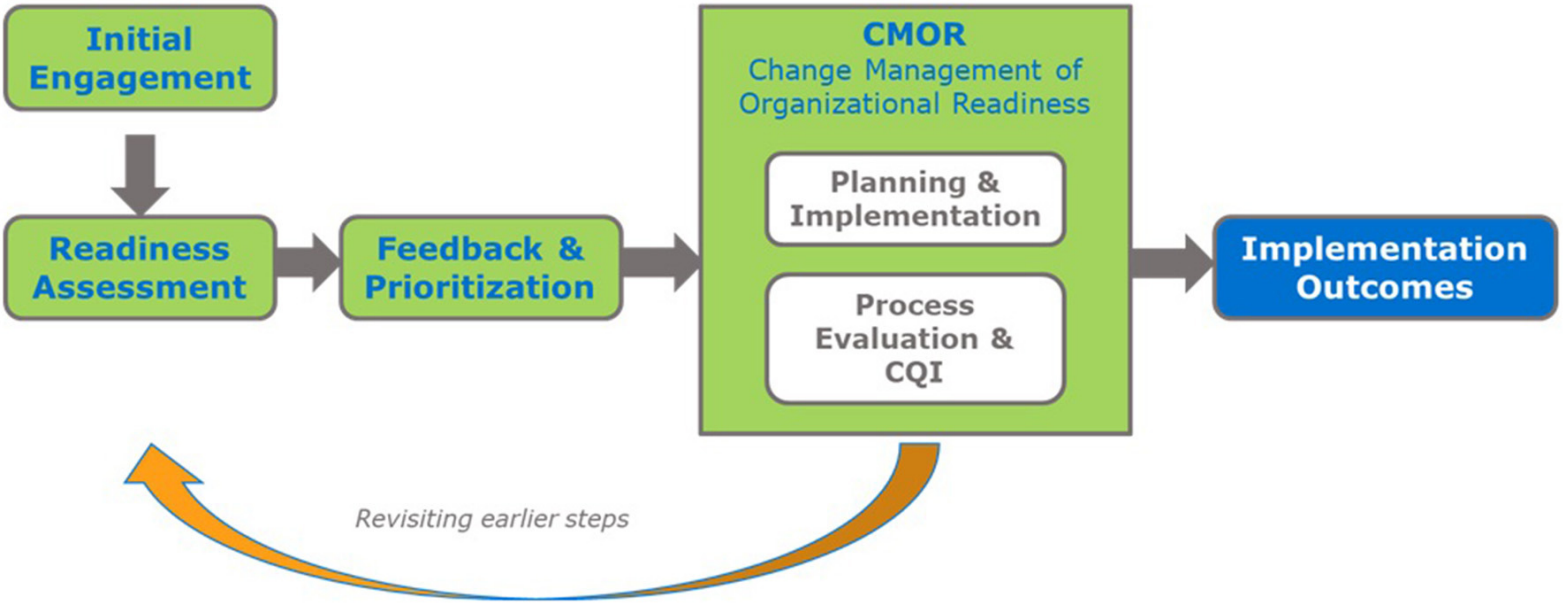
The readiness building system [RBS; (15, 16)].

Although organizational readiness is a critical factor for success, there is relatively little guidance on how to build readiness to enhance implementation. The four phases of the Readiness Building System (RBS), include the following: (1) Engagement, (2) Readiness Assessment, (3) Feedback and Prioritization, and (4) Change Management of Organizational Readiness [CMOR; Figure 2; (15, 16)]. While the RBS provides a general process for building organizational readiness and includes tools to assess and prioritize readiness constructs, it has lacked a detailed protocol for developing or selecting strategies to improve readiness. Without such guidance, an opportunity is lost; organizations may not know the specific actions (e.g., strategies) they need to employ to build their readiness. Thus, there continues to be a need for a systematic approach to building readiness. IM, which is designed to be used in conjunction with other tools and frameworks, is one protocol that can address this gap. IM provides a structured approach that systematically links readiness building strategies to the desired outcomes they are designed to influence.

### Using Implementation Mapping to Build Organizational Readiness for Sexual Assault Prevention

The Multi-Site Sexual Assault Prevention Initiative (MSSAP) is a large and long-term capacity building project taking place at eight sites across the U.S. with support from technical assistance (TA) providers. The purpose of the initiative is to increase adoption and implementation of EBIs at each site to prevent sexual assault, a serious public health problem affecting millions of men and women annually (17). To identify and adapt or develop readiness building strategies designed to improve organizational readiness, our team used RBS tools to measure and prioritize readiness subcomponents and used IM to develop and/or adapt strategies for readiness building.

**Figure 3:**
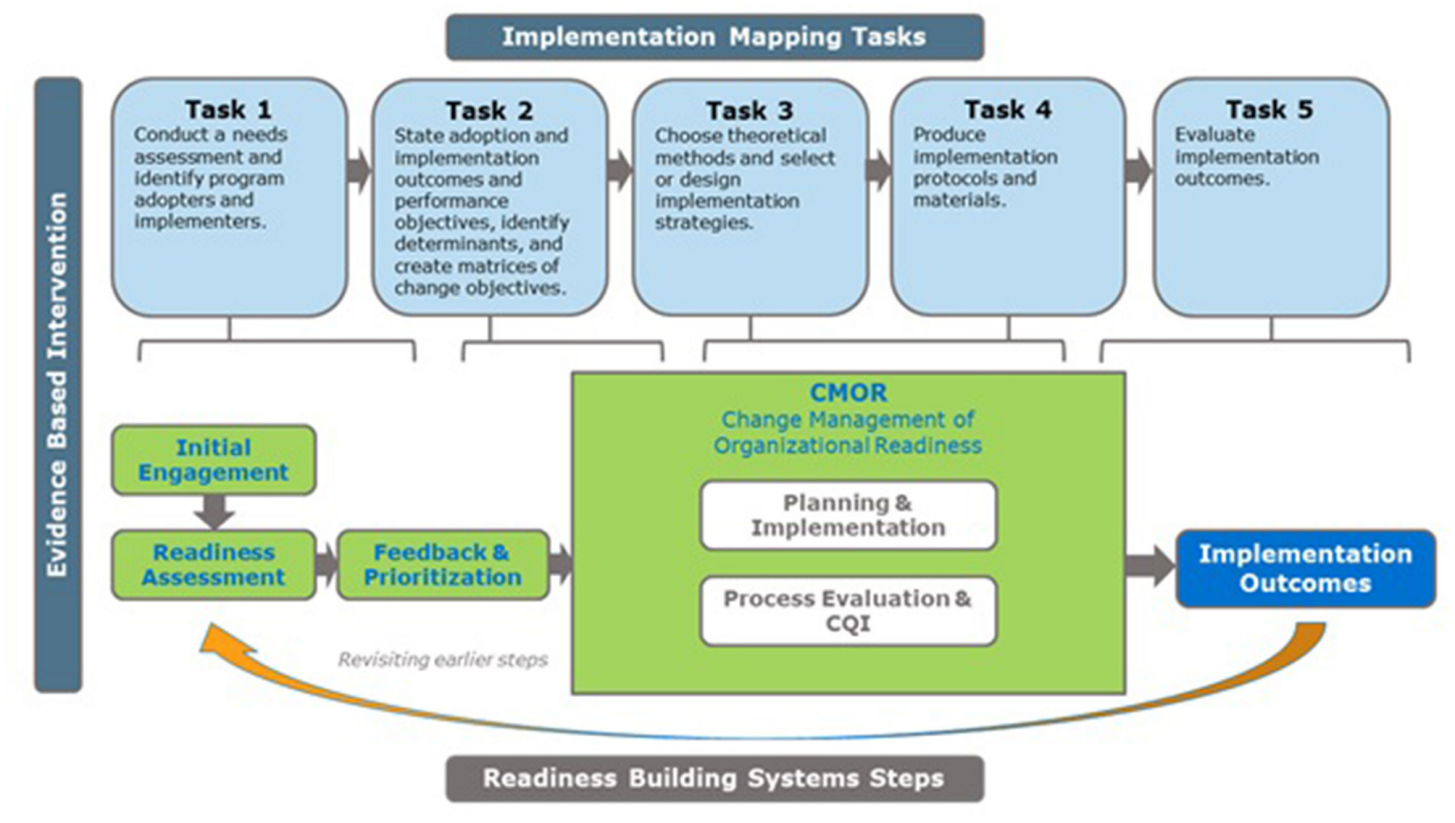
Implementation mapping and readiness building system alignment.

Figure 3 illustrates the alignment between RBS and IM. Several of the steps in both frameworks overlap. For example, the needs and assets assessment phase of IM is analogous to the engagement and assessment of organizational readiness phases of RBS. IM steps 2–4 fall within the CMOR phase of RBS. IM Steps 5 and 6 relate to evaluation and feedback to earlier phases as in RBS. In the MSSAP project, we used RBS tools for assessing and prioritizing readiness constructs to determine the most salient factors influencing implementation and IM to create the readiness building strategies. Below we describe the process we followed, highlighting examples from the MSSAP in each phase. At the time of writing this article, MSSAP was still ongoing with concurrent implementation and TA provided (specific site information is de-identified).

## Methods

As presented in Figure 3, we followed the five IM tasks with each site, which were broadly informed by the RBS: (1) conduct a needs assessment and identify program adopters and implementers; (2) state adoption and implementation outcomes and performance objectives, identify determinants, and create matrices of change objectives; (3) choose theoretical methods and select or design implementation strategies; (4) produce implementation protocols and materials; and (5) plan for evaluation of implementation outcomes (Figure 1). Across all sites, TA providers engaged partners throughout the process by conducting initial site visits, identifying stakeholders to serve as members of a worksite implementation team, participating in regularly scheduled phone calls, leading worksite implementation teams through the 5 IM tasks, and providing expertise and feedback when appropriate.

**To conduct a readiness/needs assessment (Implementation Mapping Task 1)**, an adapted Readiness Diagnostic Scale (RDS) was administered during the *Readiness Assessment Phase* of the RBS. Grounded in the *R* = MC^2^ framework, we measured organizational readiness using the RDS with response choices on a 7-point Likert scale (1 = Strongly Disagree, 7 = Strongly Agree). The scale has been used previously, and current studies are being conducted to further develop the scale and assess its psychometric properties (18, 19). Because the vast majority of sites had not selected the specific sexual assault prevention EBI to implement, the instrument was adapted to a 48-item survey that measured *general capacity* and *motivation* domains (and *not innovation-specific capacity*).

The RDS was administered electronically to implementation team members and other key informants selected by the site leadership. These respondents typically included leaders with decision-making power and those familiar with the potential barriers and facilitators to successfully implementing sexual assault prevention EBIs in their setting.

During regularly scheduled meetings via phone, worksite implementation teams and their TA providers (known together as the “implementation team”) met to discuss the results of their RDS and to work in collaboration to determine the subcomponent of readiness they wished to prioritize for readiness building efforts. The RBS provides detailed guidance on how to determine the most salient subcomponent for readiness building using a Prioritization Tool.

Once the readiness subcomponents were prioritized, the implementation teams determined adoption and implementation outcomes, stated performance objectives, identified the underlying determinant, and **created matrices for change objectives (IM Task 2)**. Theoretical methods or change mechanisms were then operationalized to **select and/or design readiness building strategies (IM Task 3)**. Implementation protocols including action plans and other **relevant materials were produced (IM Task 4)**, and the readiness building strategies were implemented. Evaluation of the strategy's implementation was conducted **and implementation outcomes were measured (IM Task 5)**.

## Results

This section describes the results of using IM to identify and develop readiness building strategies, enhanced by the incorporation of the RBS. Below, we highlight each IM task using examples from the MSSAP.

### Implementation Mapping, Task 1: Conduct a Needs Assessment and Identify Program Adopters and Implementers

IM Task 1 can be described (as shown in our alignment model; Figure 3) in three sub-tasks which correspond to three of the four RBS “phases” (Engagement, Readiness Assessment, Feedback and Prioritization).

#### Task 1a. Engagement

The TA provider engaged stakeholders who were involved in the adoption and implementation of sexual assault prevention programs at each site to participate in an implementation team. The team consisted of those in roles such as sexual assault prevention coordinators, prevention program facilitators, sexual assault victim advocates, peer support liaison personnel, equal opportunity managers, and organizational leaders. The implementation team identified areas of low readiness for implementing sexual assault prevention EBIs at the site which informed potential readiness building strategies. Additionally, at least one member from the implementation team served as the point of contact for the site and would coordinate project activities with the TA provider. Examples of TA activities included ongoing engagement, joint planning, and specific guidance for moving forward with the readiness building process conducted mainly through virtual TA.

#### Task 1b. Readiness Assessment

**Figure 4:**
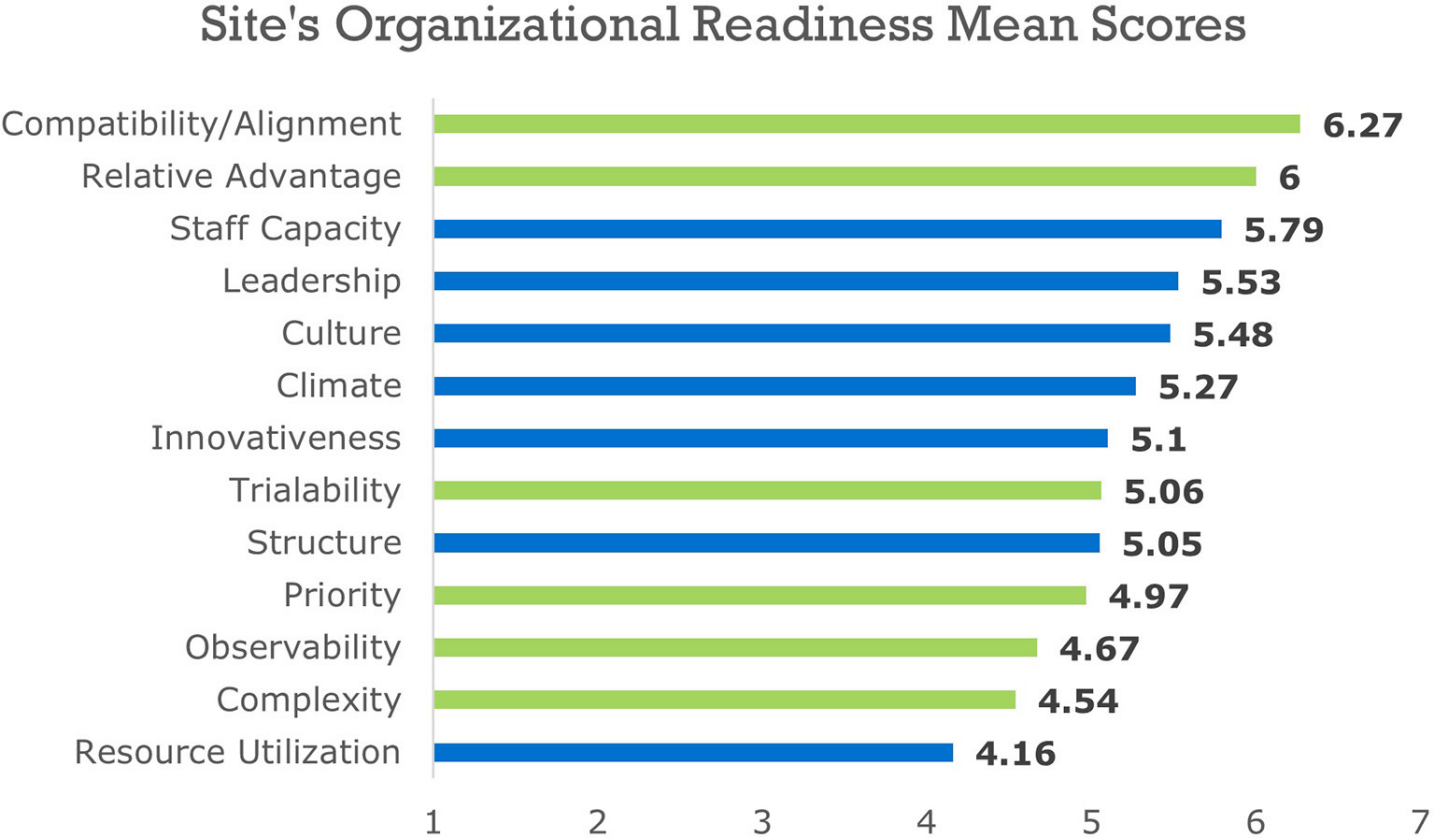
De-identified organizational readiness mean scores. Green bars are motivation subcomponents, blue bars are general capacity subcomponents.

The RDS was completed by 107 implementation team members across the eight sites with a customized Readiness Report provided to the implementation team. Data were analyzed at the organizational level and the average mean scores for each readiness subcomponent were calculated. The Readiness Reports facilitated the selection of the specific readiness components (motivation and general capacity) that were relatively stronger and weaker for each site. Figure 4 includes sample de-identified data contained in a Readiness Report. The chart displays mean organizational readiness scores for motivation subcomponents in green and general capacity subcomponents in blue. Supplemental information about the importance of the three highest and lowest readiness subcomponents was also provided in the report.

#### Task 1c. Feedback and Prioritization

As part of the needs assessment process (IM Task 1, which corresponds with the Feedback and Prioritization phase of the RBS), implementation teams identified three readiness subcomponents that they wanted to improve. The implementation teams used a Prioritization Tool to identify readiness subcomponents needing improvement based on the mean scores included in the report, the likelihood of having an impact on implementation outcomes, timeliness, priority for the change, and feasibility of the change (resources and staff are available, change is simple, etc.) (20). Because of the perceived feasibility for change, the three lowest subcomponents were not always the ones prioritized. For example, resource utilization was a subcomponent that scored relatively low for most sites; however, there was a general understanding that very little could be done to improve this subcomponent given current funding levels. Therefore, this subcomponent was documented as important, but excluded from readiness building strategy planning efforts across sites. Across the participating sites, the most common subcomponents prioritized for change were leadership, complexity, priority, and observability.

### Implementation Mapping, Task 2: State Adoption and Implementation Outcomes and Performance Objectives, Identify Determinants, and Create Change Matrices of Change Objectives

Task 2 (as shown in Figure 3), as well as Tasks 3 and 4, correspond to the *CMOR* phase section of the RBS framework. Using IM, implementation teams were able identify factors influencing the various readiness subcomponents needing attention and develop approaches to address them.

The implementation teams progressed to Task 2 after identifying the prioritized subcomponents for change. The readiness building outcomes for each prioritized subcomponent were identified by answering the question: “What needs to change related to [subcomponent] to improve the site's organizational readiness?” Examples of readiness building outcomes included: “The worksite will make sexual assault prevention a priority,” “The mid- and senior-level leaders will actively support sexual assault prevention programming,” and “Implementers will assess the short-term outcomes of the program to increase observability.” The performance objectives, which are sub-tasks needed to achieve the implementation outcomes, were determined by answering the question: “Who needs to do what in order to achieve the improvements in the readiness component, and, in turn, implementation outcome?” Examples of performance objectives included: “The prevention coordinators will communicate success stories from the pilot test with Leadership,” “The prevention coordinators will cultivate appropriate working relationships,” and “Leadership displays commitment and involvement in the implementation of sexual assault prevention programs.”

The implementation teams identified determinants of the readiness building outcomes by using dissemination, implementation, and behavioral health theories and frameworks, empirical evidence, and input from the implementation team at each site. Examples of determinants include attitudes toward sexual assault prevention, attitudes about and awareness of the specific sexual assault prevention EBI, the program specific knowledge, self-efficacy, and skills, the perception of risk associated with not performing implementation behaviors, and the outcome expectations of the sexual assault prevention EBI.

Matrices of change objectives were created by crossing each of the determinants with performance objectives and answering: “What needs to change in the determinant for the implementer to accomplish the performance objective?” Examples of change objectives and the associated performance objectives are included in the partial sample matrix shown in Table 3. Matrices of change objectives were created for each subcomponent within general capacity and motivation (*N* = 13) and formed the blueprint for identifying and developing implementation strategies to improve readiness (Task 3).

**Table 3:** Partial matrix of change for observability (subcomponent of motivation).

**Performance objectives**	**Attitudes/awareness**	**Self-efficacy**	**Knowledge**	**Skills**	**Outcome expectations**
A. Prevention coordinators will assess the short-term impact of the sexual assault prevention program among participants (*Observability*).	AA1. Prevention Coordinators believe that assessing short-term impact of the sexual assault prevention program has advantages. AA2. Prevention Coordinators believe that assessing short-term impact of the sexual assault prevention program should be a priority. AA3. Prevention Coordinators believe that assessing short-term impact of the sexual assault prevention program is simplistic. AA4. Prevention Coordinators believe that the sexual assault prevention program fits the needs of the target population.	ASE1. Prevention coordinators express confidence in their ability to assess the short-term impact of the sexual assault prevention program among participants. ASE2. Prevention coordinators express confidence in their ability to assess and analyze data. ASE2. Prevention Coordinators express confidence in their ability to reach short-term outcomes.	AK1. Prevention coordinators identify short-term outcome measures for the sexual assault prevention program. AK2. Prevention Coordinators list characteristics of the sexual assault prevention program. AK3. Prevention Coordinators describe the support needed to assess the short- term impact of the sexual assault prevention program.	AS1. Prevention coordinators demonstrate their evaluation plan for assessing the short-term impact of the sexual assault prevention program. AS2. Prevention coordinators demonstrate ability to implement metrics to measure short-term impacts of the sexual assault prevention program.	AOE1. Prevention coordinators believe that assessing short-term outcomes will help improve the success of the implementation of sexual assault prevention programs. AOE2. Prevention Coordinators believe that the sexual assault prevention program will lead to outcomes. AOE3. Prevention Coordinators believe that the sexual assault prevention program will help meet organizational priorities. AOE3. Prevention Coordinators believe that the assessment of outcomes from the sexual assault prevention program will be successfully sustained over time.

### Implementation Mapping, Task 3: Choose Theoretical Methods and Select or Design Implementation Strategies

To select, adapt, or develop the readiness building strategies that would achieve the readiness building outcome, implementation teams identified theoretical methods known to target the determinants identified (and associated with the specific change objectives within the matrices as outlined in Task 2). Theoretical methods are a key component of the mechanisms of action for influencing determinants, while practical applications of these methods, described here as readiness building strategies, operationalize them in a way that is consistent with the population and setting (10, 21). After methods to influence change in the determinants were identified, each implementation team developed specific strategies to operationalize these methods and ensured that the strategies developed were feasible to implement. To save time and resources, when possible, we leveraged and enhanced existing strategies that were being implemented at each site. For example, the performance objective “Leadership displays commitment and involvement in the implementation of sexual assault prevention programs” and it's associated change objective “Leaders believe that displaying commitment and involvement for programs is a priority,” can be influenced by the change methods of arguments, persuasive communication, and repeated exposure. To operationalize these methods in one site, one site selected to distribute fact sheets that highlight the prevalence and organizational consequences (e.g., reduced productivity, mental health burden, etc.) of sexual assault. These fact sheets were regularly distributed to mid-level leaders prior to each time the sexual assault program was implemented.

Because each site (1) prioritized different readiness subcomponents, (2) implemented different sexual assault prevention EBIs, and (3) had varying levels of resources available for implementation, there was no standardized set of readiness building strategies that were used across all sites. Rather, each site identified specific strategies that targeted the readiness subcomponent they had prioritized for their site. Examples of readiness building strategies are included in Table 4. The change objectives are listed with corresponding theoretical change methods and specific strategies.

**Table 4:** Example change methods and readiness building strategies and their associated change objectives.

**Change objectives for worksite A**	**Determinants**	**Change methods**	**Parameters**	**Readiness building strategies**
AA1. Prevention Coordinators believe that assessing short-term impact of the sexual assault prevention program has advantages. AA2. Prevention Coordinators believe that assessing short-term impact of the sexual assault prevention program should be a priority. ASE1. Prevention Coordinators express confidence in their ability to assess the short-term impact of the sexual assault prevention program among participants. AOE1. Prevention Coordinators believe that assessing short-term outcomes will help improve the success of the implementation of sexual assault prevention programs.	Attitudes, self-efficacy, and outcome expectations	A. Guided practice B. Discussion C. Feedback	A. Sub-skill demonstration, instruction, and enactment with Individual feedback; requires supervision by an experienced person; some environmental changes cannot be rehearsed. B. Listening to the learner to ensure that the correct schemas are activated. C. Feedback needs to be individual, follow the behavior in time, and be specific.	A. Technical assistance provider lead discussion and assisted implementation team in develop an implementation plan for adoption and implementation of the sexual assault prevention program. B. At monthly meeting, TA providers discuss implementation plans and outcome and process evaluation instruments. C. At monthly meeting, TA providers give feedback on implementation plans and outcome and process evaluation instruments.

### Implementation Mapping, Task 4: Produce Implementation Protocols and Materials

The implementation team adapted or developed the materials and protocols for the readiness building strategies in close collaboration with each site's implementation team. In the example with the change objective, “Leaders believe that displaying commitment and involvement for programs is a priority,” and the selected strategy of regularly distributing fact sheets, Task 4 includes the actual creation and/or editing of the fact sheets. Monthly meetings with each site were held to elicit feedback on the strategies; revisions were made accordingly. Detailed action plans were created for each readiness building strategy to outline associated tasks/materials needed, who was responsible for each, and deadlines for completion. Knowing who was responsible and when action items would be completed helped TA providers track readiness building strategy implementation across sites.

### Implementation Mapping, Task 5: Evaluate Implementation Outcomes

Task 5 in IM is used to evaluate the implementation outcomes related to program implementation. Evaluation of program implementation is currently ongoing. However, to gain an understanding of the influence of the readiness building strategy on determinants and implementation performance objectives, participating implementation teams created evaluation plans aimed at evaluating the implementation of the readiness building strategy. This included an assessment of the reach, responsiveness, and fidelity of each readiness building strategy to be implemented. Reach was defined as the number of individuals who “received the strategy,” responsiveness was defined as the degree of engagement from individuals who “received the strategy” (not engaged, semi-engaged, engaged), and fidelity was defined whether the strategy was implemented as it was planned (yes/no). To date, each site implemented between 3 and 11 readiness building-strategies with evaluation ongoing.

## Discussion

This article describes how IM and RBS were used together to develop readiness building strategies to improve organizational motivation and capacity to implement sexual assault prevention programs and therefore implementation outcomes. While the initial step of IM provides overall guidance about assessing needs and resources available for an implementation effort, RBS specifically focuses on the concept of organizational readiness (according to the *R* = MC^2^ heuristic) and includes tools to help assess and prioritize subcomponents of organizational readiness. On the other hand, while RBS provides general guidance about addressing identified readiness building-needs through “change management,” it provided relatively little guidance about how to choose and adapt or develop strategies once specific readiness needs were identified. IM addressed this gap. This article showcases how using RDS can improve the identification and prioritization of factors that need to be addressed to improve organizational readiness and, thus, implementation. IM provides guidance about what to do with this information through a step-by-step process for developing readiness building strategies to improve implementation of evidence-based interventions.

A strength of this study is that it addresses an ongoing challenge in implementation science: identifying and tailoring the most appropriate implementation strategies to address identified barriers (7). Although several methods have been proposed to improve the systematic selection or development of implementation strategies, few provide a process that explicitly maps strategies to needs and simultaneously guides the development of concrete change objectives and content that enable that change. While IM has been used for the development of, or selection and tailoring of, implementation strategies for a variety of topics and settings, this is the first time it was used to build readiness for sexual assault prevention. Additionally, this is the first time it has been used to develop *readiness building strategies* specifically designed to increase organizational readiness. Researchers and practitioners agree that organizational readiness is important for successful implementation; systematic approaches guided by theory and evidence to inform the selection of methods and strategies that will impact specific determinants of implementation are needed (1–4, 7). Without approaches that use logic, evidence, theory, and systematic processes to incorporate these into decisions about strategy selection and tailoring, the use of strategies to build readiness will continue to be left to best guesses.

In the examples presented, we described the process of how the RBS and IM were used to develop strategies to improve readiness for the implementation of sexual assault prevention EBIs. Initially, we used RBS tools for assessing and prioritizing readiness subcomponents, we then used IM to identify performance objectives and determinants of readiness outcomes. IM then guided the selection of change techniques (methods) and specific site-appropriate strategies to build readiness (readiness-building strategies). This approach was used with eight different sites implementing programs to prevent sexual assault.

Community and stakeholder engagement in implementation science has received significant attention over the years and engagement of a broad array of stakeholders is needed to understand what is required for successful implementation (including what makes an organization ready to implement) and how to accelerate and improve the process (22). Both the RBS and IM underscore the importance of community and stakeholder engagement and provide explicit directions for how to engage the implementation team to develop implementation strategies during the needs and resources assessment phase and during the selection and tailoring of readiness building strategies. For the participating sites, the feedback and prioritization component continued in an iterative manner throughout the strategy development process. The RBS provided the tools for assessing and prioritizing readiness and the understanding that readiness building is an iterative process, and IM provided a structured way to engage with stakeholders by guiding teams through specific tasks. The IM tasks provide a natural structure to inform planning sessions with stakeholders while also allowing for iterative changes as the team learns what is needed to build and sustain readiness. However, the sites were not explicitly taught these processes. Rather, sites received TA to guide them through the process. TA providers used specific questions to identify readiness outcomes, performance objectives, and underlying determinants. In the future, additional user-friendly tools and a manual will likely need to be developed and distributed to guide sites through this process without the presence of intensive TA supports.

## Limitations

While the project and the process described has many strengths, there are a number of limitations. First, sites were at varying stages in the process of identifying a sexual assault prevention program to implement. There are few sexual assault EBIs for the specific population of focus that have been well-researched (23–26). Therefore, there was variability in their ability to define barriers and facilitators of implementation of “sexual assault prevention” generally rather than considering a specific program. As a result, several sites had not selected a program by the time that readiness building activities began. Therefore, it made little sense to assess and/or prioritize “innovation specific” readiness subcomponents. Thus, this important component of readiness was not assessed formally at the beginning of the project. Nevertheless, since general capacity and motivation are likely prerequisites to implement *any* sexual assault prevention program, addressing these subcomponents is likely to contribute to positive outcomes. To ensure readiness, as sites selected a program, they received TA to informally assess “innovation-specific readiness” and followed a similar approach to build innovation-specific capacity.

Another challenge was the ability to sustain the intensive efforts of planning and implementing a new program during the COVID-19 pandemic. The pandemic required significant modifications, including changing expectations and timelines. This often delayed and/or extended the TA being provided.

## Conclusion

Organizational readiness is a critical factor for implementing EBIs, but there is little guidance on how to improve it. Using the RBS with IM is one approach to build an organization's readiness to adopt and implement EBIs. Using these frameworks synergistically provides a systematic process to further articulate the barriers to implementation, craft readiness goals and outcomes, identify determinants of readiness that can be addressed, and select and tailor readiness-building strategies. Future research should focus on the utility of using the RBS in conjunction with IM to develop readiness-building strategies, as well as evaluating the impact of these strategies on implementation outcomes.

## Data Availability Statement

The datasets presented in this article are not readily available due to protection of privacy. Requests for data can be directed to AWat, awatson@wandersmancenter.org.

## Ethics Statement

The studies involving human participants were reviewed and approved by RAND IRB. Written informed consent for participation was not required for this study in accordance with the national legislation and the institutional requirements.

## Author Contributions

AWat, BH, JK-G, TW, and MF all wrote sections of the manuscript. All authors contributed to conception and design of study and contributed to manuscript revision, read, and approved the final submission.

## Funding

This work was supported by the National Heart, Lung, and Blood Institute (K01HL151817 to TW) and National Cancer Institute (1R01CA228527-01A1).

## Author Disclaimer

The content is solely the responsibility of the authors and does not necessarily represent the official views of the National Institutes of Health, National Cancer Institute, or the RAND Corporation.

## Conflict of Interest

The authors declare that the research was conducted in the absence of any commercial or financial relationships that could be construed as a potential conflict of interest.

## Publisher's Note

All claims expressed in this article are solely those of the authors and do not necessarily represent those of their affiliated organizations, or those of the publisher, the editors and the reviewers. Any product that may be evaluated in this article, or claim that may be made by its manufacturer, is not guaranteed or endorsed by the publisher.
